# The BSA-induced Ca(2+) influx during sperm capacitation is CATSPER channel-dependent

**DOI:** 10.1186/1477-7827-7-119

**Published:** 2009-10-27

**Authors:** Jingsheng Xia, Dejian Ren

**Affiliations:** 1 Department of Biology, University of Pennsylvania, 415 S University Ave, Philadelphia, Pennsylvania 19104, USA

## Abstract

**Background:**

Serum albumin is a key component in mammalian sperm capacitation, a functional maturation process by which sperm become competent to fertilize oocytes. Capacitation is accompanied by several cellular and molecular changes including an increased tyrosine phosphorylation of sperm proteins and a development of hyperactivated sperm motility. Both of these processes require extracellular calcium, but how calcium enters sperm during capacitation is not well understood.

**Methods:**

BSA-induced changes in intracellular calcium concentration were studied using Fluo-4 and Fura-2 calcium imaging with wild-type and Catsper1 knockout mouse sperm.

**Results:**

We found that the fast phase of the BSA-induced rises in intracellular calcium concentration was absent in the Catsper1 knockout sperm and could be restored by an EGFP-CATSPER1 fusion protein. The calcium concentration increases were independent of G-proteins and phospholipase C but could be partially inhibited when intracellular pH was clamped. The changes started in the principal piece and propagated toward the sperm head.

**Conclusion:**

We conclude that the initial phase of the increases in intracellular calcium concentration induced by BSA requires the CATSPER channel, but not the voltage-gated calcium channel. Our findings identify the molecular conduit responsible for the calcium entry required for the sperm motility changes that occur during capacitation.

## Background

During mammalian fertilization, freshly ejaculated sperm do not have the ability to fertilize oocytes until after they undergo capacitation, a functionally defined, but poorly understood maturation process by which sperm become capable of fertilizing eggs [1-3]. Sperm become capacitated *in vivo*, by interacting with environmental stimuli in the female reproductive tract before encountering eggs. This process can also be mimicked *in vitro *by incubating sperm in defined capacitation media. Several commonly used components are essential for successful *in vitro *capacitation in sperm from many mammalian species. Among them are bovine serum albumin (BSA), Ca^2+ ^and bicarbonate (HCO_3_^-^) [3]. Capacitation leads to several cellular and behavioral changes, including an increase in tyrosine phosphorylation of sperm proteins, rises in intracellular pH (pH_i_) and Ca^2+ ^concentration ([Ca^2+^]_i_), membrane hyperpolarization, and hyperactivated motility [4-6].

Increases in [Ca^2+^]_i _and intracellular [pH]_i_ are believed to play central roles in both sperm capacitation and the acrosome reaction (AR) [3,7,8]. The capacitating agent BSA induces Ca^2+ ^influx in sperm, but the molecular mechanisms underlying such an influx are not well understood. Multiple Ca^2+^-permeable ion channels have been detected in mammalian sperm, including voltage-gated Ca^2+ ^channels (Ca_V_s), transient receptor potential (TRP) channels, cyclic nucleic gated (CNG) channels and CATSPER channels [9-12]. Among these ion channel proteins, only the four mammalian CATSPER members (CATSPER 1-4) are specifically found in sperm and spermatogenic cells [13-17]. All four *Catsper *genes are required for male fertility as mice with any of these genes disrupted are infertile [15,18-20]. Disruptions in *Catsper1* and *Catsper2* are also associated with male infertility in humans [21-23].

Using Ca^2+^-sensitive fluorescent probes, we and others have shown that CATSPERs are required for the Ca^2+ ^entry induced by stimuli such as cyclic nucleotides, alkaline depolarizing medium and egg coat proteins [24-26]. Ca^2+ ^entering the channel in sperm tail can trigger Ca^2+ ^propagation toward the head [25,26]. CATSPER's roles in the migration of sperm toward the oocyte and in penetrating the egg coat have been clearly established by studies showing that *Catsper *mutant sperm cannot migrate to the egg *in vivo *[27] and that, in *in vitro *fertilization (IVF), they cannot penetrate coat-intact eggs but can fertilize those without the zona pellucida [18]. In contrast, CATSPER's function in sperm capacitation is less clear. *Catsper *mutant sperm do not develop hyperactive motility after incubation in capacitation medium, as do normal sperm. The mutant sperm also have a progressive decrease of motility under certain incubation conditions [14,15,19,24,28,29]. This finding suggests that CATSPER has a role in the motility aspect of sperm capacitation. On the other hand, wild-type and *Catsper *mutant sperm do not differ in their patterns of protein tyrosine phosphorylation after sperm capacitation [19,24] or in their capacitation and AR efficiency, as examined with the chlortetracycline (CTC) assay [25,26]. In this study, we investigated CATSPER's potential role in sperm capacitation by studying the Ca^2+ ^influx induced by BSA.

## Methods

### Reagents

Fluo-4 AM, Fura-2 AM and pluronic F-127 were purchased from Molecular Probes (Invitrogen, Eugene, OR). Pertussis toxin (PTX) and ionomycin were from CalBiochem (Gibbstown, NJ) and Cell-Tak was from BD Biosciences (Bedford, MA). BSA (fraction V, fatty acid-depleted, Sigma #A3059), disodium salt ATP, and other reagents were purchased from Sigma. Similar Ca^2+ ^responses in sperm were also observed with fatty acid-free BSA (Sigma #A8806; not shown).

### Animals

Animals were treated according to institutional regulations. This study used *Catsper1 *knockout mice that were backcrossed to C57BL/6J for more than 10 generations [18]. Sperm of the *Catsper1 *knockout mice lack not only CATSPER1, but also CATSPER2 [28] and the associated auxiliary proteins CATSPERβ [16] and CATSPERγ [17]. To reflect this fact, we do not distinguish CATSPER1 from the other CATSPERs throughout the paper. The *EGFP*-*Catsper1 *transgenic mice have a *Catsper1 *null background but carry an EGFP-CATSPER1 fusion protein gene that rescues the male sterile phenotype of the *Catsper1 *null mutant [16].

### Sperm Ca^2+ ^imaging

Non-capacitated caudal sperm were used for Ca^2+ ^imaging, as previously described [25,26]. Briefly, sperm were released into HS medium containing (mM): 135 NaCl, 5 KCl, 2 CaCl_2_, 1 MgCl_2_, 30 HEPES, 10 glucose, 10 lactic acid, and 1 pyruvic acid (pH adjusted to 7.4 with NaOH), and concentrated to 5 × 10^6 ^-1 × 10^7^/ml. Cells were loaded with 10 μM Fluo-4 AM and 0.05% pluronic F-127 for 30 min at room temperature in the dark, followed by two washes in imaging medium (HS supplemented with 15 mM NaHCO_3_), each with a 4 min spin at 300 × *g*. Washed sperm were resuspended in imaging medium and loaded into a small-volume imaging chamber (~1 cm diameter, ~90 μl) formed with Sylgard on a Cell-Tak coated coverslip, and allowed to attach for ~10 min.

For imaging sperm from *EGFP- Catsper1 *transgenic mice, a ratiometric measurement with Fura-2 (5 μM for loading) was used because of the EGFP fluorescence. A monochromator (DeltaRAM V, PTI) with a 75-W Xenon lamp was used to generate the excitation at 488 nm for Fluo-4 (or 340 nm and 380 nm for Fura-2). A 60× objective and a 1.6× adaptor on an inverted microscope (IX-71, Olympus) were used for imaging. Emissions (515-565 nm) were bandpass filtered (HQ540/50, Chroma) and collected with a cooled CCD camera (CoolSNAP HQ, Roper Scientific) for 25 ms in every 0.5 s for fast recording, or 100 ms in every 6 s for slow recording. Online control, data collection, and image processing were conducted using commercial software (ImageMaster 3, PTI).

For imaging using Fluo-4, [Ca^2+^]_i _changes are presented as ΔF/F_0 _ratios after background subtraction, where ΔF was the change in fluorescence signal intensity and F_0 _was the baseline as calculated by averaging the 10 frames before stimulus application. In sperm loaded with Fura-2, [Ca^2+^]_i _changes are presented as the ratio of fluorescence from excitation at 340 nm to that at 380 nm (F340/F380) after background subtraction. All the imaging experiments were done at room temperature. Cells with uneven dye loading were excluded from the analysis. Motile sperm (~60% of the population) that had at least two points attached to the coverslip were used for analysis. Cells with peak changes of >50% in ΔF/F_0 _(for Fluo-4) or >0.1 in F340/380 (for Fura-2) after application of stimuli were counted as responsive. To detect the Ca^2+ ^responses at "clamped" membrane potential (V_m_), K^+ ^ionophore valinomycin (1 μM) was added to the imaging buffer, with additional K^+ ^as indicated to replace an equimolar amount of Na^+^. E_K _was calculated with the assumption of a 120 mM intracellular K^+ ^concentration [30,31]. To "clamp" intracellular pH, sperm were preincubated for 5 min with 3 μM carbonylcyanide-p-trifluoromethoxyphenyl hydrazone (FCCP) and 1 μM valinomycin [32].

### Statistical methods

Data analyses were performed with ImageMaster3, Excel and Origin. Student's t-tests and ANOVA were used for statistical comparisons between different treatment groups. P < 0.05 was considered statistically significant.

## Results

### CATSPER channels are required for the BSA-induced [Ca^2+^]_i _rise in mouse sperm

**Figure 1:**
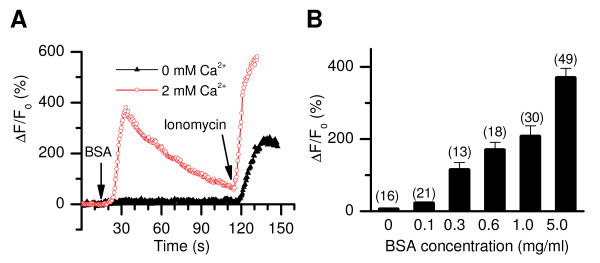
**BSA-induced [Ca^2+^]_i _changes in the sperm head of wild-type mice monitored with single cell imaging**. (*A*) Representative recordings of [Ca^2+^]_i _changes (represented as normalized Fluo-4 fluorescence changes) in response to application of BSA (5 mg/ml; indicated by vertical arrow) to a bath containing 2 mM Ca^2+ ^or no Ca^2+ ^(with 5 mM EGTA). Some cells also had a second phase of [Ca^2+^]_i _rise (see Figure 2A). Ionomycin (5 μM, used as a control) caused a [Ca^2+^]_i _rise in the absence of extracellular Ca^2+ ^by releasing intracellular Ca^2+^. (*B*) Dose -dependence of peak amplitude of the BSA-induced Ca^2+ ^responses.

When applied to non-capacitated sperm lightly immobilized onto coverslips, BSA elevated [Ca^2+^]_i _in the sperm head (Figure 1A) at concentrations as low as 0.1 mg/ml (Figure 1B). The responses were dependent on the presence of extracellular Ca^2+ ^(Figure 1A), suggesting a role for Ca^2+ ^entry. These properties of BSA-induced changes under our conditions are comparable with other studies in mouse and human sperm [33-35].

**Figure 2:**
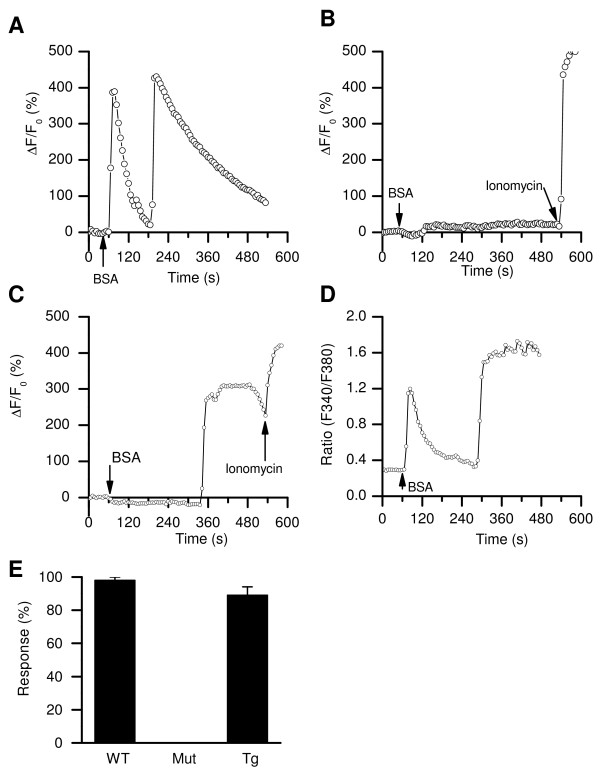
**BSA-induced [Ca^2+^]_i _rises in sperm head depend on CATSPER**. (*A*) Example of a wild-type sperm with two phases of [Ca^2+^]_i _changes. (*B*, *C*) Recordings from *Catsper1 *null sperm. None had the fast (1^st ^phase) response within 2 min, but one (*C*) had a 2^nd ^phase response. The Ca^2+ ^ionophore ionomycin (5 μM) was applied as a control stimulus. (*D*) A representative recording from a transgenic sperm expressing an EGFP-CATSPER fusion protein in the *Catsper1 *null background. (*E*) Percentage of sperm responsive to BSA application in wild-type (WT; n = 18 imaging runs; 9 mice, 109 cells), *Catsper1 *null (Mut; n = 7; 3 mice, 30 cells), and a *Catsper1 *mutant rescued with the EGFP-CATSPER transgene (Tg; n = 12; 5 mice, 57 cells). Cells from Tg mice were imaged with Fura-2 and the others were imaged with Fluo-4. BSA was used at a concentration of 5 mg/ml.

How BSA induces [Ca^2+^]_i _changes in sperm is not well understood, but one possibility is through Ca_V_s [34-36]. Our recent studies, however, suggest that mature sperm do not have detectable functional Ca_V _channels [25]. To determine whether the BSA-induced [Ca^2+^]_i _rises are dependent on CATSPERs, we compared the [Ca^2+^]_i _changes in wild-type mouse sperm with those in the Catsper1 null mutants. Upon bath application of BSA (5 mg/ml), 98% of wild-type sperm (107/109) showed initial responses in the head within 20 s and 17% (10 of 58) had a 2nd response more than 2 min later (Figure 2A, E). In contrast, the initial [Ca^2+^]_i _changes were absent in CATSPER1-deficient sperm within 2 min of BSA stimulation (Figure 2B, E), although the delayed responses were present in some sperm (23%, Figure 2C). The BSA-induced [Ca^2+^]_i _rises were restored by a transgene encoding an EGFP-CATSPER1 fusion protein in the Catsper1 null background (Figure 2D, E). These results indicate that CATSPER1 is required for the initial [Ca^2+^]_i _responses.

### Elevations in BSA -induced [Ca^2+^]_i _start in the sperm tail and propagate to the head

**Figure 3:**
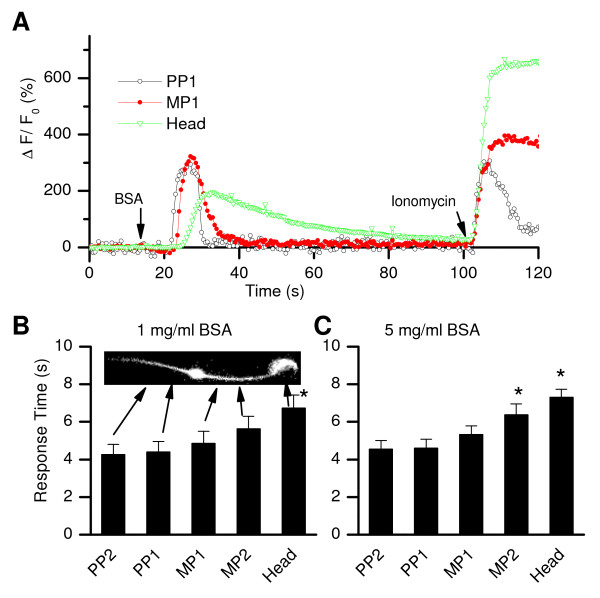
**The BSA -induced [Ca^2+^]_i _rises start in sperm tail and propagate toward the head**. (*A*) Representative time courses of the [Ca^2+^]_i _changes in the principal piece (PP1), midpiece (MP1) and head of the sperm. Increases in [Ca^2+^]_i _start in the principal piece and propagate to the head. The Ca^2+ ^ionophore ionomycin (5 μM) induced [Ca^2+^]_i _increases simultaneously in all subregions. (*B*-*C*) Time differences between the application of BSA (*B*, 1 mg/ml; *C*, 5 mg/ml) and the onset of fluorescence changes (defined as the time point when ΔF/F_0 _started to have steep rise) in different regions within the principal piece (PP1, PP2), midpiece (MP1, MP2) and head as shown in the inset. PP1 and MP1 are both 5 μm from the annulus. There is a 5 μm distance from PP1 to PP2, and from MP1 to MP2. * indicates statistically significant (P < 0.05).

The finding that the BSA-induced increase in [Ca^2+^]_i _in the sperm head was dependent on CATSPER1 was intriguing because CATSPER proteins and the current through the channels are strictly localized in sperm principal piece, which is 20 mm away from the head [18,37]. To test whether the BSA-induced [Ca^2+^]_i _changes in the sperm head were a result of Ca^2+ ^entry through CATSPER in the tail, we analyzed the spatial-temporal kinetics of [Ca^2+^]_i _changes along the entire length of the sperm. After BSA application, [Ca^2+^]_i _rises started in the principal piece, and then were seen in the mid-piece and head of the sperm (Figure 3A). The differences in the response onsets between the principal piece and head were 2.46 ± 0.52 s (n = 10) with 1 mg/ml BSA (Figure 3B) and 2.76 ± 0.25 s (n = 10) when induced with a higher concentration (5 mg/ml, Figure 3C). In contrast to BSA, the Ca^2+^ ionophore ionomycin (5 μM) increased [Ca^2+^]_i _simultaneously in the principal piece, mid-piece and sperm head (Figure 3A).

### The BSA-induced increases in [Ca^2+^]_i _partially depend on pH_i _changes

**Figure 4:**
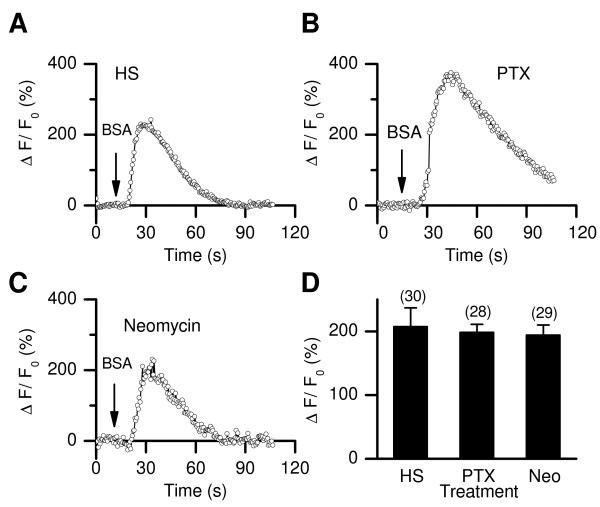
**PTX and neomycin do not inhibit the BSA-induced [Ca^2+^]_i _change**. (*A*-*C*) Representative recordings of the [Ca^2+^]_i _changes (ΔF/F_0_) in the sperm head induced by BSA (1 mg/ml) from cells pre-incubated with control (*B*), PTX (100 ng/ml, 30 min pre-incubation) or neomycin (*C*) (1 mM, 5 min pre-incubation). (*D*) Averaged peak ΔF/F_0 _changes. The numbers of total sperm (from two to three mice) are indicated.

How does BSA induce a CATSPER-dependent Ca^2+ ^entry? Stimuli such as egg coat proteins (e.g. zona pellucida) can lead to CATSPER-dependent Ca^2+ ^influx likely via pertussis toxin (PTX)-sensitive G proteins and a phospholipase C-dependent signaling pathway that can be inhibited by neomycin [10,25,38]. To test whether the CATSPER-dependent Ca^2+ ^influx activated by BSA uses a similar pathway, we recorded BSA-induced [Ca^2+^]_i _changes in the absence and presence of PTX (100 ng/ml, Figure 4B) or neomycin (1 mM, Figure 4C). Neither PTX or neomycin inhibited the BSA-induced [Ca^2+^]_i _rises (Figure 4D).

**Figure 5:**
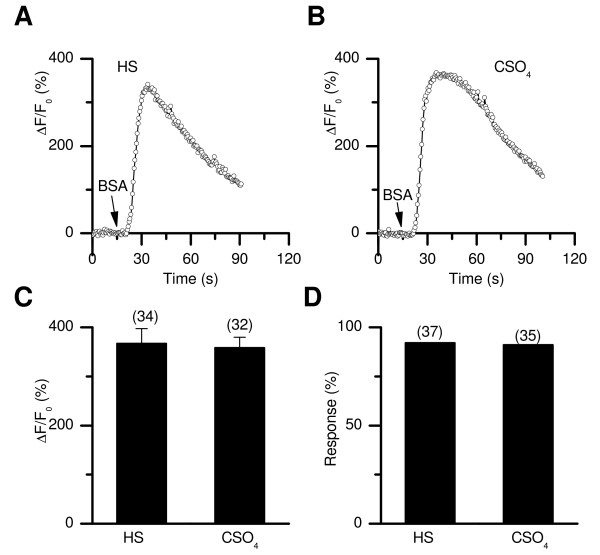
**BSA-induced [Ca^2+^]_i _changes in the presence and absence of cholesterol**. (*A*, *B*) Representative [Ca^2+^]_i _rises induced by BSA (5 mg/ml) in the absence (*A*) and presence (*B*) of CSO_4 _(preincubated with 750 μM CSO_4 _for 30 min). (*C*, *D*) Averaged peak ΔF/F_0 _changes (*C*) and percentages of cells responsive to BSA in the absence and presence of CSO_4_.

BSA also changes the membrane lipid composition by facilitating cholesterol efflux, and such a change likely contributes to capacitation-associated events such as tyrosine phosphorylation [39-42]. The ability of BSA to remove lipids, however, does not seem to be required for its induction of CATSPER-dependent Ca^2+ ^influx since pre-incubating sperm with cholesterol sulfate to presumably saturate BSA and reduce its ability to remove cholesterol [36,41] did not inhibit the BSA-induced Ca^2+ ^influx (Figure 5).

**Figure 6:**
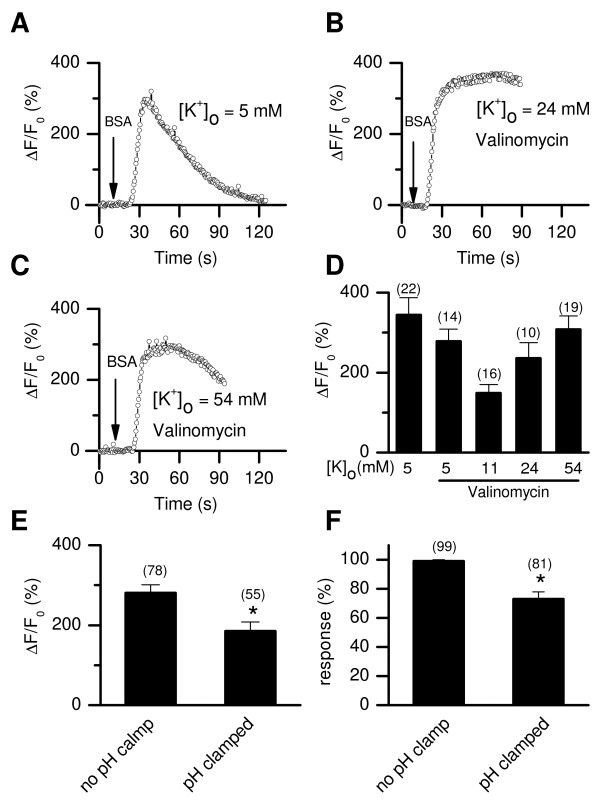
**BSA-induced [Ca^2+^]_i _rises under clamped membrane potential (V_m_) or intracellular pH (pH_i_)**. (*A*-*C*) Representative recordings in different [K^+^]_o _as indicated, without (*A*) or with (*B *and *C*) 1 μM valinomycin in the bath to "clamp" the membrane potentials to -40 mV ([K^+^]_o _= 24 mM) or -20 mV ([K^+^]_o _= 54 mM). (*D*) Averaged peak ΔF/F_0 _in responsive cells under various [K^+^]_o_, with or without valinomycin. (*E*, *F*) Peak [Ca^2+^]_i _rise amplitude (*E*) and response percentages (*F*) induced by 5 mg/ml BSA without pH_i _clamp or under pH_i _clamp. Numbers of tested cells are indicated within the bars. *, P < 0.05 compared to the no pH_i _clamp condition.

Depolarization-activated voltage-gated channels such as the T-type Ca^2+^ channel have also been proposed to mediate the BSA-induced [Ca^2+^]_i _increases [34-36]. In addition, CATSPER channels are not primarily voltage-activated, but do have some weak voltage-sensitivity [37]. To test whether voltage changes are required for the BSA-induced Ca2+ entry into mature sperm, we compared [Ca^2+^]_i _changes at various membrane potentials "clamped" with a K^+^ ionophore valinomycin. Although the amplitudes of the [Ca^2+^]_i _change varied with the clamped membrane potentials, BSA still raised [Ca^2+^]_i _even when the voltage of the membrane was held at -40 (Figure 6B, D) or -20 mV (Figure 6C, D), conditions that are expected to completely inactivate the T-type Ca2+ channel[25]. These data suggest that voltage changes nor T-type Ca^2+ ^channels are required for the BSA-induced elevations in [Ca^2+^]_i_.

Another effect of BSA in particular, and capacitation in general, is an intracellular alkalization by 0.4 pHi units [33,43]. When pH_i_ was clamped with a H^+^ ionophore FCCP and K^+^ ionophore valinomycin [32], the amplitude of the BSA-induced [Ca^2+^]_i _rise was decreased and the percentage of responsive cells was reduced (Figure 6E, F), suggesting that BSA induces [Ca^2+^]_i _increases, at least partially, through pHi changes.

**Figure 7:**
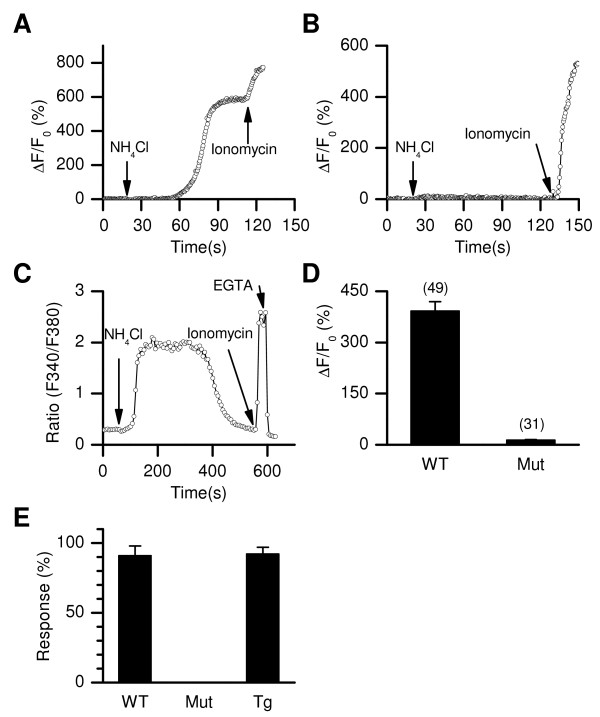
**CATSPER1 is required for the NH_4_Cl-induced [Ca^2+^]_i _rise in mouse sperm**. (*A*-*C*) Representative responses to 20 mM NH_4_Cl in sperm head from a wild-type (*A*), a *Catsper1 *null (*B*), and transgenic sperm expressing an EGFP-CATSPER1 fusion protein on the *Catsper1 *null background (*C*). Ionomycin (10 μM, *A*-*C*) and EGTA (50 mM) were applied as control stimuli. (*D*) Averaged ΔF/F_0 _changes induced by 20 mM NH_4_Cl in wild-type (WT) and *Catsper1 *null (Mut) sperm. Numbers of analyzed cells are in parentheses. (*E*) Percentages of sperm responsive to NH_4_Cl application in WT (n = 7 imaging runs; 3 mice, 56 cells), *Catsper1 *null (Mut; n = 7; 3 mice, 31 cells), and the *Catsper1 *mutant rescued with the *EGFP-CatSper1 *transgene (Tg; n = 7; 2 mice, 31 cells). Cells from Tg mice were imaged with Fura-2 and the others were imaged with Fluo-4.

Consistent with a role of pH_i _changes in the BSA-induced [Ca^2+^]_i _increases, the CATSPER channel can be activated by intracellular alkalization [37] and alkaline medium with an elevated concentration of K^+ ^(the K8.6 medium) can lead to a CATSPER-dependent rise in [Ca^2+^]_i _[24]. Thus, we investigated whether intracellular alkalization alone can induce CATSPER-dependent [Ca^2+^]_i _rises and the extent to which CATSPER channels contribute to the increases. To do this, we compared the [Ca^2+^]_i _responses to bath application of 20 mM NH_4_Cl, which leads to intracellular alkalization and induces a slow [Ca^2+^]_i _rises in sperm [44-47], in the wild-type and Catsper1 mutant sperm. NH4Cl application evoked [Ca^2+^]_i _rises in wild-type, but not in *Catsper1* null sperm (Figure 7). The NH_4_Cl-induced [Ca^2+^]_i _changes were restored by an EGFP-CATSPER1 fusion protein in the Catsper1 null background (Figure 7C, E). These results suggest that the CATSPER channel is necessary for the increase in [Ca^2+^]_i _induced by pHi change.

### The ATP-induced [Ca^2+^]_i _rises in mouse sperm are independent of CATSPER channel

**Figure 8:**
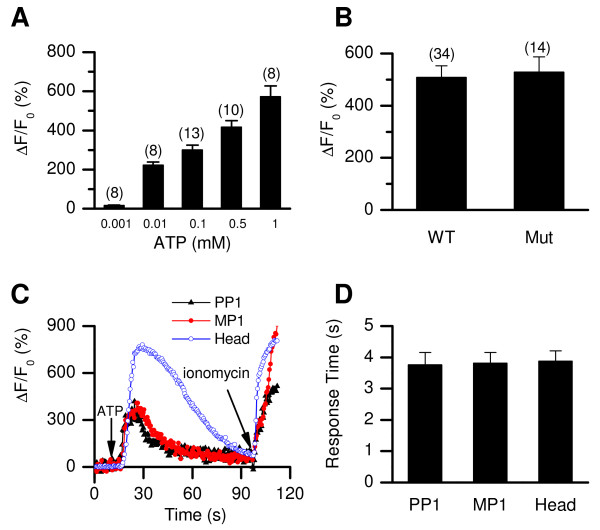
**CATSPER channels are not required for the ATP-induced [Ca^2+^]_i _rise**. (*A*) Dose-responses of ATP-induced [Ca^2+^]_i _rises in the sperm head of wild-type (WT) mouse. (*B*) Peak amplitudes of ΔF/F_0 _induced by 0.1 mM ATP in WT and *Catsper1 *null (Mut) sperm. (*C*) Representative recordings of the time course of ATP (1 mM) -induced fluorescence changes in the principal piece (PP1), midpiece (MP1) and head, in a WT sperm. Like the ones induced by Ca^2+ ^ionophore (10 μM ionomycin), the ATP-elicited [Ca^2+^]_i _increases started simultaneously in all subregions (*D*, n = 8). The locations of the subregions were defined as in Figure 3.

The findings that *Catsper1* mutant sperm lack the initial [Ca^2+^]_i _increased induced by several stimuli tested (8-Br-cGMP, 8-Br-cAMP, alkaline depolarizing medium, zona pellucida, pH_i_ change (NH_4_Cl) and BSA [24-26]) raises a concern that a deficiency in the CATSPER channel leads to a non-specific defect in sperm Ca^2+^ entry. To test this idea, we compared the Catsper1 mutant and wild-type's sperm [Ca^2+^]_i _responses to bath application of ATP, which was reported to increase [Ca^2+^]_i _in bovine and mouse sperm by activating a P2 purinergic receptor[48,49]. Similar to other observations, ATP induced rises in [Ca^2+^]_i _in the wild-type sperm head in a concentration-dependent manner (Figure 8A). Unlike the BSA-induced [Ca^2+^]_i _increases, those induced by ATP did not begin in the tail, but instead, started simultaneously along the whole length of the sperm (Figure 8C, D). The ATP-induced [Ca^2+^]_i _changes were intact in the Catsper null sperm (Figure 8B), indicating that the CATSPER channel does not contribute to the ATP-induced elevation of [Ca^2+^]_i_.

## Discussion

The major finding of our study is that CATSPER channels are required for the BSA induced [Ca^2+^]_i _increases in mouse sperm. Stimuli known to induce a CATSPER-dependent Ca^2+ ^entry now include some of the most important mediators in sperm physiology: cyclic nucleotides, zona pellucida and serum albumin. There are clearly also CATSPER-independent Ca^2+ ^entry paths that are responsible for the [Ca^2+^]_i _increases induced by stimuli such as extracellular ATP, as we showed in this study, and mechanical force (Xia and Ren, unpublished). Differences in signal transduction pathways that link various stimuli to CATSPER channels also exist. For example, the Ca^2+ ^entry induced by the zona pellucid is dependent on G-proteins and phospholipase C [10,25,38] whereas the BSA-induced one is not (Figure 4).

Like several other stimuli such as 8-Br-cGMP [37] and the zona pellucida [25], BSA does not directly activate CATSPER channel currents in corpus sperm under whole cell voltage clamp patch clamp recording (with intracellular and extracellular pH buffered at 7.2 and 7.4, respectively [25,37]; Xia and Ren, unpublished observation). We cannot exclude the possibility that BSA can directly activate CATSPER under more physiological conditions. During sperm capacitation with BSA-containing medium, there is an intracellular alkalization [43]. Consistent with a role of the alkalization, the BSA-induced [Ca^2+^]_i _rises were reduced when pH_i _was "clamped" (Figure 6). The [Ca^2+^]_i _response, however, was not completely inhibited. The residual BSA-induced [Ca^2+^]_i _changes under "pH_i _clamp" condition are likely through pathways other than intracellular alkalization.

Although weakly voltage-dependent, whole cell CATSPER conductance can be increased by cell membrane depolarization [37]. In addition, depolarization by increased extracellular K^+ ^concentrations, especially coupled with alkalization medium (K8.6), can lead to a CATSPER-dependent Ca^2+ ^influx [24]. These results point to a possible role of membrane depolarization in the pathway leading to the BSA-induced Ca^2+ ^entry. However, we consider this possibility unlikely for several reasons. First, we show here that the [Ca^2+^]_i _rises are not dependent on a membrane voltage change since they persist when the voltage is "clamped" with K^+ ^ionophore. Second, inactivating the T-type Ca_V _channel, that is a proposed candidate for the Ca^2+ ^entry [34-36], by clamping the membrane at -20 mV does not decrease the BSA-induced [Ca^2+^]_i _rises. This result is consistent with our recent finding that mature sperm do not possess detectable functional Ca_V _channels [25]. Finally, BSA does not lead to depolarization during sperm capacitation, instead, a hyperpolarization of ~15 mV has been observed using voltage-sensitive fluorescence dyes [31]. Along these lines, we used whole cell patch clamp under current clamp mode to directly measure acute membrane potential changes in corpus sperm upon BSA application and found that BSA (4 mg/ml) produced a fast and profound membrane hyperpolarization (11 ± 2 mV, n = 6). While the exact mechanism of such a membrane potential hyperpolization induced by BSA remains undetermined, we found no evidence for a role of membrane depolarization in the BSA-induced Ca^2+ ^entry through CATSPER.

## Conclusion

In summary, we determined the molecular identity of an ion channel responsible for the fast Ca^2+ ^influx induced by BSA used in *in vitro *sperm capacitation. Three lines of evidence support that CATSPER is the aforementioned channel. First, the BSA-induced [Ca^2+^]_i _increases are absent in *Catsper1 *knockout sperm; second, such responses can be restored by an EGFP-CATSPER1 fusion protein; and finally, these [Ca^2+^]_i _rises start in the principal piece of sperm where CATSPER proteins and current through CATSPER channels are localized. The mechanisms by which BSA is coupled to the CATSPER channel are largely unknown. They are unlikely through a voltage change or a cholesterol efflux. An intracellular alkalization appears to be involved (Figure 6) but there is clearly a [pH]_i _change-independent component that remains to be uncovered.

Together with previous findings that the capacitation status and changes in the pattern of tyrosine phosphorylation during capacitation do not require CATSPER while the hyperactivated motility does [15,20,24,28], our data genetically separate the Ca^2+ ^requirements for the two aspects of sperm capacitation: one mediating the change of motility via the CATSPER channel and another mediating the increase of tyrosine phosphorylation via a source yet to be determined. One possible source for the latter is the second phase of [Ca^2+^]_i _rises that are also present in the *Catsper1 *mutant sperm.

## Competing interests

The authors declare that they have no competing interests.

## Authors' contributions

JX performed all the experiments and did data analysis. JX and DR designed the experiments and wrote the paper. Both authors read and approved the final manuscript.

## References

[B1] Chang MC (1951). Fertilizing capacity of spermatozoa deposited into the fallopian tubes. Nature.

[B2] Austin CR (1952). The capacitation of the mammalian sperm. Nature.

[B3] Yanagimachi R, Knobil E, Neill JD (1994). Mammalian Fertilization. The Physiology of Reproduction.

[B4] Bedu-Addo K, Lefievre L, Moseley FL, Barratt CL, Publicover SJ (2005). Bicarbonate and bovine serum albumin reversibly 'switch' capacitation-induced events in human spermatozoa. Mol Hum Reprod.

[B5] Suarez SS (2008). Control of hyperactivation in sperm. Hum Reprod Update.

[B6] Visconti PE (2009). Understanding the molecular basis of sperm capacitation through kinase design. Proc Natl Acad Sci USA.

[B7] Breitbart H (2002). Intracellular calcium regulation in sperm capacitation and acrosomal reaction. Mol Cell Endocrinol.

[B8] Florman HM, Jungnickel MK, Sutton KA (2008). Regulating the acrosome reaction. Int J Dev Biol.

[B9] Darszon A, Acevedo JJ, Galindo BE, Hernandez-Gonzalez EO, Nishigaki T, Trevino CL, Wood C, Beltran C (2006). Sperm channel diversity and functional multiplicity. Reproduction.

[B10] Florman HM, Arnoult C, Kazam IG, Li C, O'Toole CM (1998). A perspective on the control of mammalian fertilization by egg- activated ion channels in sperm: a tale of two channels. Biol Reprod.

[B11] Jimenez-Gonzalez C, Michelangeli F, Harper CV, Barratt CL, Publicover SJ (2005). Calcium signalling in human spermatozoa: a specialized 'toolkit' of channels, transporters and stores. Hum Reprod Update.

[B12] Wassarman PM, Jovine L, Litscheer ES (2001). A profile of fertilization in mammals. Nature Cell Biology.

[B13] Lobley A, Pierron V, Reynolds L, Allen L, Michalovich D (2003). Identification of human and mouse CatSper3 and CatSper4 genes: Characterisation of a common interaction domain and evidence for expression in testis. Reprod Biol Endocrinol.

[B14] Jin JL, O'Doherty AM, Wang S, Zheng H, Sanders KM, Yan W (2005). Catsper3 and catsper4 encode two cation channel-like proteins exclusively expressed in the testis. Biol Reprod.

[B15] Qi H, Moran MM, Navarro B, Chong JA, Krapivinsky G, Krapivinsky L, Kirichok Y, Ramsey IS, Quill TA, Clapham DE (2007). All four CatSper ion channel proteins are required for male fertility and sperm cell hyperactivated motility. Proc Natl Acad Sci USA.

[B16] Liu J, Xia J, Cho KH, Clapham DE, Ren D (2007). Catsper beta: A novel transmembrane protein in the catsper channel complex. J Biol Chem.

[B17] Wang H, Liu J, Cho KH, Ren D (2009). The novel single-transmembrane protein CATSPERG is associated with mouse CATSPER1 channel protein. Bio Reprod.

[B18] Ren D, Navarro B, Perez G, Jackson AC, Hsu S, Shi Q, Tilly JL, Clapham DE (2001). A sperm ion channel required for sperm motility and male fertility. Nature.

[B19] Quill TA, Sugden SA, Rossi KL, Doolittle LK, Hammer RE, Garbers DL (2003). Hyperactivated sperm motility driven by CatSper2 is required for fertilization. Proc Natl Acad Sci USA.

[B20] Jin J, Jin N, Zheng H, Ro S, Tafolla D, Sanders KM, Yan W (2007). Catsper3 and Catsper4 are essential for sperm hyperactivated motility and male fertility. Biol Reprod.

[B21] Avidan N, Tamary H, Dgany O, Cattan D, Pariente A, Thulliez M, Borot N, Moati L, Barthelme A, Shalmon L, Krasnov T, Ben-Asher E, Olender T, Khen M, Yaniv I, Zaizov R, Shalev H, Delaunay J, Fellous M, Lancet D, Beckmann JS (2003). CATSPER2, a human autosomal nonsyndromic male infertility gene. Eur J Hum Genet.

[B22] Zhang Y, Malekpour M, Al-Madani N, Kahrizi K, Zanganeh M, Mohseni M, Mojahedi F, Daneshi A, Najmabadi H, Smith RJH (2007). Sensorineural deafness and male infertility: a contiguous gene deletion syndrome. J Med Genet.

[B23] Avenarius M, Hildebrand M, Zhang Y, Meyer N, Smith L, Kahrizi K, Najmabadi H, Smith R (2009). Human male infertility caused by mutations in the CATSPER1 channel protein. Am J Hum Genet.

[B24] Carlson AE, Westenbroek RE, Quill T, Ren D, Clapham DE, Hille B, Garbers DL, Babcock DF (2003). CatSper1 required for evoked Ca^2+ ^entry and control of flagellar function in sperm. Proc Natl Acad Sci USA.

[B25] Xia J, Ren D (2009). Egg-coat proteins activate calcium entry into mouse sperm via CATSPER channels. Biol Reprod.

[B26] Xia J, Reigada D, Mitchell CH, Ren D (2007). CATSPER channel-mediated Ca^2+ ^entry into mouse sperm triggers a tail-to-head propagation. Biol Reprod.

[B27] Ho K, Wolff CA, Suarez SS (2009). CatSper-null mutant spermatozoa are unable to ascend beyond the oviductal reservori. Reprod Fertil Dev.

[B28] Carlson AE, Quill TA, Westenbroek RE, Schuh SM, Hille B, Babcock DF (2005). Identical phenotypes of CatSper1 and CatSper2 null sperm. J Biol Chem.

[B29] Marquez B, Ignotz G, Suarez SS (2007). Contributions of extracellular and intracellular Ca^2+ ^to regulation of sperm motility: Release of intracellular stores can hyperactivate CatSper1 and CatSper2 null sperm. Dev Biol.

[B30] Demarco IA, Espinosa F, Edwards J, Sosnik J, De La Vega-Beltran JL, Hockensmith JW, Kopf GS, Darszon A, Visconti PE (2003). Involvement of a Na^+^/HCO_3_^- ^cotransporter in mouse sperm capacitation. J Biol Chem.

[B31] Zeng Y, Clark EN, Florman HM (1995). Sperm membrane potential: hyperpolarization during capacitation regulates zona pellucida-dependent acrosomal secretion. Dev Biol.

[B32] Christen R, Schackmann RW, Shapiro BM (1983). Metabolism of sea urchin sperm. J Biol Chem.

[B33] Huang YH, SP K, MH L, Shih CM, Chu ST, Wei CC, Wu TJ, Chen YH (2005). Signals of seminal vesicle autoantigen suppresses bovine serum albumin-induced capacitation in mouse sperm. Biochem Biophys Res Commun.

[B34] Blackmore PF, Eisoldt S (1999). The neoglycoprotein mannose-bovine serum albumin, but not progesterone, activates T-type calcium channels in human spermatozoa. Mol Hum Reprod.

[B35] Sakata Y, Saegusa H, Zong S, Osanai M, Murakoshi T, Shimizu Y, Noda T, Aso T, Tanabe T (2002). Ca_V_2.3 (alpha1E) Ca^2+ ^channel participates in the control of sperm function. FEBS Lett.

[B36] Espinosa F, I Lo-Ga, Munoz-Garay C, Felix R, De la Vega-Beltran JL, Kopf GS, Visconti PE, Darszon A (2000). Dual regulation of the T-type Ca^2+ ^current by serum albumin and beta- estradiol in mammalian spermatogenic cells. FEBS Lett.

[B37] Kirichok Y, Navarro B, Clapham DE (2006). Whole-cell patch clamp measurements of spermatozoa reveal an alkaline-activated Ca^2+ ^channel. Nature.

[B38] Fukami K, Yoshida M, Inoue T, Kurokawa M, Fissore RA, Yoshida N, Mikoshiba K, Takenawa T (2003). Phospholipase Cdelta4 is required for Ca^2+ ^mobilization essential for acrosome reaction in sperm. J Cell Biol.

[B39] Wolf DE, Hagopain SS, Ishijima S (1986). Changes in sperm plasma membrane lipid diffusibility after hyperactivation during in vitro capacitation in the mouse. J Cell Biol.

[B40] Osheroff JE, Visconti PE, Valenzuela JP, Travis AJ, Alvarez J, Kopf GS (1999). Regulation of human sperm capacitation by a cholesterol efflux-stimulated signal transduction pathway leading to protein kinase A-mediated up-regulation of protein tyrosine phosphorylation. Mol Hum Reprod.

[B41] Visconti PE, Ning X, Fornes MW, Alvarez JG, Stein P, Connors SA, Kopf GS (1999). Cholesterol efflux-mediated signal transduction in mammalian sperm: cholesterol release signals an increase in protein tyrosine phosphorylation during mouse sperm capacitation. Dev Biol.

[B42] Go KJ, Wolf DE (1985). Albumin-mediated changes in sperm sterol content during capacitation. Biol Reprod.

[B43] Zeng Y, Oberdorf JA, Florman HM (1996). pH regulation in mouse sperm: identification of Na^+^-, Cl^-^, and HCO_3_^-^-dependent and arylaminobenzoate-dependent regulatory mechanisms and characterization of their roles in sperm capacitation. Dev Biol.

[B44] Wennemuth G, Westenbroek RE, Xu T, Hille B, Babcock DF (2000). Ca_V_2.2 and Ca_V_2.3 (N- and R-type) Ca^2+ ^channels in depolarization- evoked entry of Ca^2+ ^into mouse sperm. J Biol Chem.

[B45] Santi CM, Santos T, Hernandez-Cruz A, Darszon A (1998). Properties of a novel pH-dependent Ca^2+ ^permeation pathway present in male germ cells with possible roles in spermatogenesis and mature sperm function. J Gen Physiol.

[B46] Fraire-Zamora JJ, Gonzalez-Martinez MT (2004). Effect of intracellular pH on depolarization-evoked calcium influx in human sperm. Am J Physiol Cell Physiol.

[B47] Marquez B, Suarez SS (2007). Bovine Sperm Hyperactivation Is Promoted by Alkaline-Stimulated Ca^2+ ^Influx. Biol Reprod.

[B48] Luria A, Rubinstein S, Lax Y, Breitbart H (2002). Extracellular adenosine triphosphate stimulates acrosomal exocytosis in bovine spermatozoa via P2 purinoceptor. Biol Reprod.

[B49] Rodríguez-Miranda E, Buffone M, Edwards S, Ord T, Lin K, Sammel M, Gerton G, Moss S, Williams C (2008). Extracellular adenosine 5'-triphosphate alters motility and improves the fertilizing capability of mouse sperm. Biol Reprod.

